# Case Report: Difficulties in diagnosis of myeloid sarcoma of the breast by core needle biopsy

**DOI:** 10.3389/fmed.2025.1633268

**Published:** 2025-11-26

**Authors:** Demian Wu, Xiaolan Li, Xiaoxue Tian, Ting Xu, Qiyao Ge, Shuai Luo, Jinjing Wang

**Affiliations:** 1 Department of Pathology, Affiliated Hospital of Zunyi Medical University, Zunyi, Guizhou, China; 2 Class 3, Clinical Medicine, Grade 2023, Guizhou Medical University, Guiyang, Guizhou, China

**Keywords:** myeloid sarcoma, the mammary gland, acute myeloid leukemia, pathology, treatment

## Abstract

**Background:**

Myeloid sarcoma (MS) is a rare neoplasm that arises from myeloid blasts outside the bone marrow. Diagnosing and treating MS in the breast can be challenging due to its rarity in this location. Clinicians should consider MS when a breast mass is detected to implement appropriate treatment and avoid unnecessary surgery.

**Case demonstration:**

A 25-year-old female with a 2-year history of acute myeloid leukemia and a 1-year history of hematopoietic stem cell transplantation was admitted due to bilateral breast masses present for 1 month. Breast MRI plain scan + enhancement revealed bilateral breast masses with irregular shapes, irregular borders, and significant enhancement, suggesting a malignant tumor. A bilateral breast biopsy was performed, and the pathological diagnosis confirmed MS involving the breast. Following the diagnosis, local radiotherapy was performed, and no recurrence was observed during the 1-month follow-up.

**Conclusion:**

The clinical manifestations of MS in the breast are typically nonspecific, and its imaging features resemble those of breast cancer or other malignant tumors. Therefore, diagnosing primary MS of the breast without a history of leukemia is challenging, and the final diagnosis requires a histopathological biopsy. For patients with a history of AML, imaging should be regularly reviewed for early detection, diagnosis, and treatment to improve prognosis.

## Background

Myeloid sarcoma (MS), also known as granulocytic sarcoma (GS), chloroma, or extramedullary myeloid tumor, is a mass formed by myeloid blasts outside the bone marrow (1). Although MS can occur in any part of the body and is most commonly found in bones, lymph nodes, soft tissues, and skin, it accounts for only 3% of breast tissue cases (2). MS in the breast is often secondary to relapse following bone marrow transplantation (BMT) in patients with acute myeloid leukemia (AML) (3). Because of the rarity of breast MS, its diagnosis and treatment are challenging. Clinicians should consider this possibility when a breast mass is detected to ensure appropriate treatment and avoid unnecessary surgeries, such as mastectomy.

### Case demonstration

A 25-year-old female was diagnosed with AML 2 years ago. She presented with symptoms of bleeding and infection, including fever, sore throat, and oral bleeding. Blasts-myeloblast/promonocyte cells accounted for 81%, suggesting a diagnosis of M4/M5 AML. Karyotype analysis showed 46, XX, inv.(16) (p13q22). Gene mutation analysis of 248 myeloid-related genes identified a missense mutation C in the *FLT3* gene of Asp835val (heterozygous, 37.2%). Fusion gene monitoring was positive for *CBFβ-MYH11*. In addition, 35G > A(p. Gly12Asp) (low frequency heterozygous, mutation frequency 1.4%, sequencing depth 3,205×) in the *NRAS* gene was detected. Based on these findings, the patient was diagnosed as AML with *CBFβ-MYH11* positivity, *FLT3-TKD*, *NRAS* gene mutation. After the first course of standard Homoharringtonine and Ara-C chemotherapy regimen(HA) chemotherapy, complete remission (CR) was achieved. She then received regular high-dose cytarabine consolidation therapy. She underwent hematopoietic stem cell transplantation in our hospital 1 year ago and recovered well after the procedure. She is currently taking ruxolitinib phosphate tablets 5 mg bid orally to manage the graft-versus-host disease (GVHD).

The patient was admitted to the hospital with a complaint of “bilateral breast masses found for 1 month.” The bilateral breast lumps had been noted a month earlier, without any obvious cause, and there was no tenderness, nipple bleeding, or discharge. The patient did not experience symptoms such as chills, fever, fatigue, poor appetite, low-grade fever, night sweats, chest tightness, chest pain, or dyspnea.

Physical examination revealed suspicious masses in both breasts, approximately 30 mm in size on the left side and 60 mm on the right side, with unclear boundaries. There was no depression in the bilateral nipples, scarring, redness, swelling, ulceration, varicosity, “orange peel sign,” and “dimple sign” on the local skin.

Breast ultrasound revealed the following findings: (1) bilateral breast hypoechoic area, BI-RADS 4a, and a biopsy was recommended; and (2) enlarged right axillary lymph nodes: reactive hyperplasia was considered.

**Figure 1:**
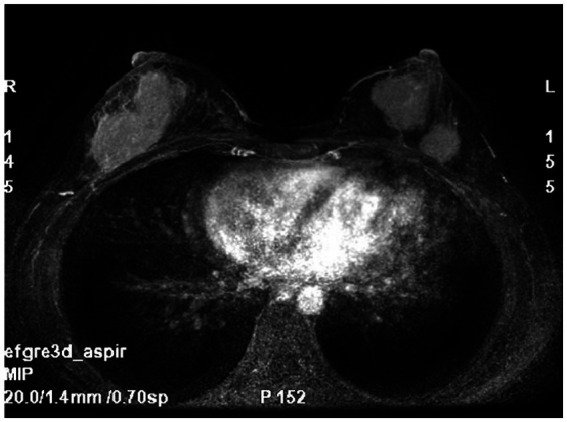
Breast MRI: bilateral breast mass, irregular shape, irregular edge, marked enhancement.

Breast MRI (Figure 1) with plain scan and enhancement revealed bilateral breast findings of type C, with low background enhancement, In the left breast, two masses with long T1 and short T2 signals were observed, both irregular in shape. The largest mass measured approximately 27 mm × 26 mm × 28 mm, with irregular edges and marked enhancement after enhancement. There was an increase in and thickening of surrounding blood vessels. The time signal curve was type II, and limited diffusion was noted. In the lower outer quadrant of the right breast, a quadrantally distributed enhancement lesion was seen, measuring approximately 61 mm × 30 mm, with increased and thickened surrounding blood vessels.

As the nature of the breast mass was uncertain, bilateral breast needle biopsy was performed and histopathological examination was performed.

**Figure 2:**
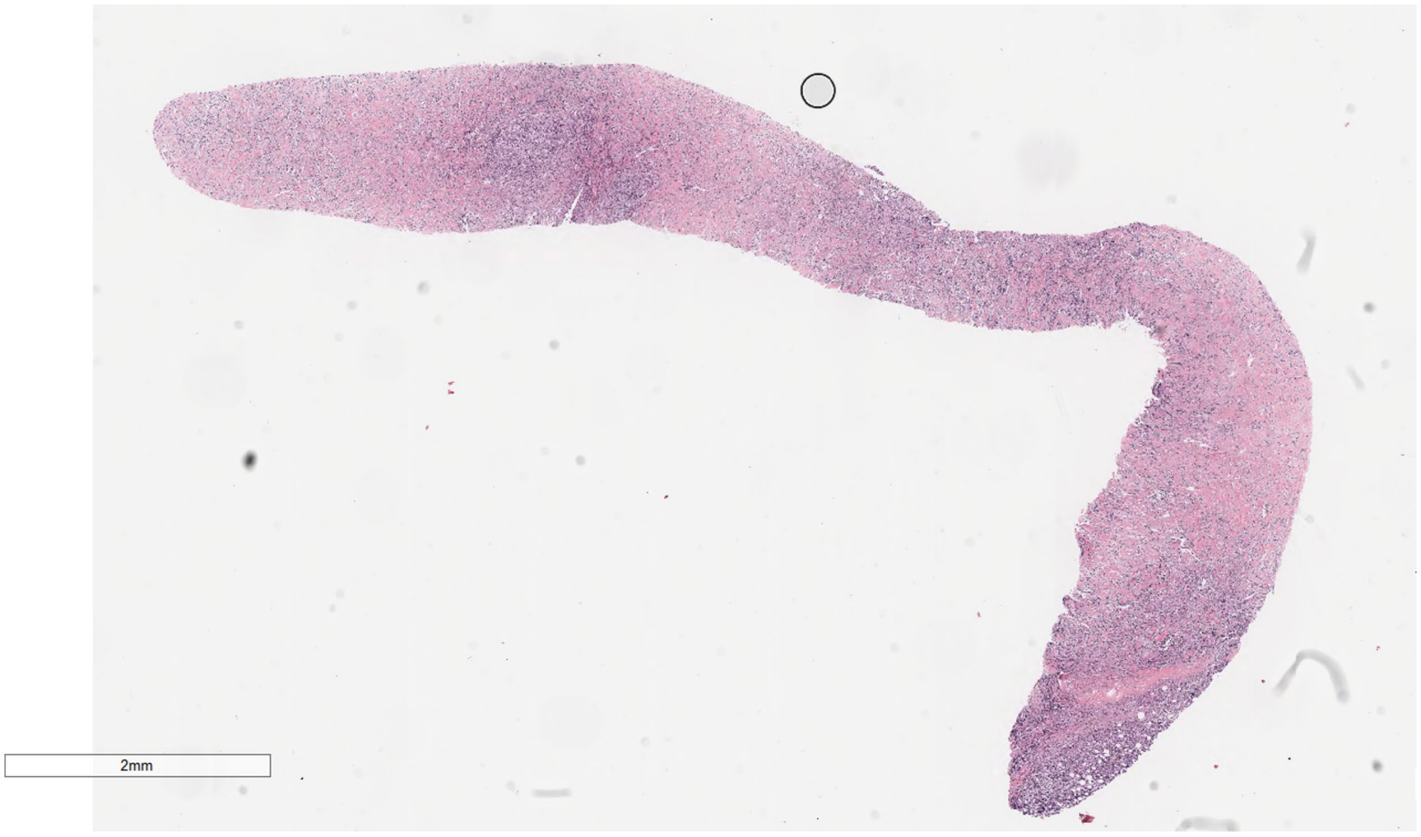
Biopsy tissue of the breast at low magnification, showing no obvious lobular structure of the breast.

**Figure 3:**
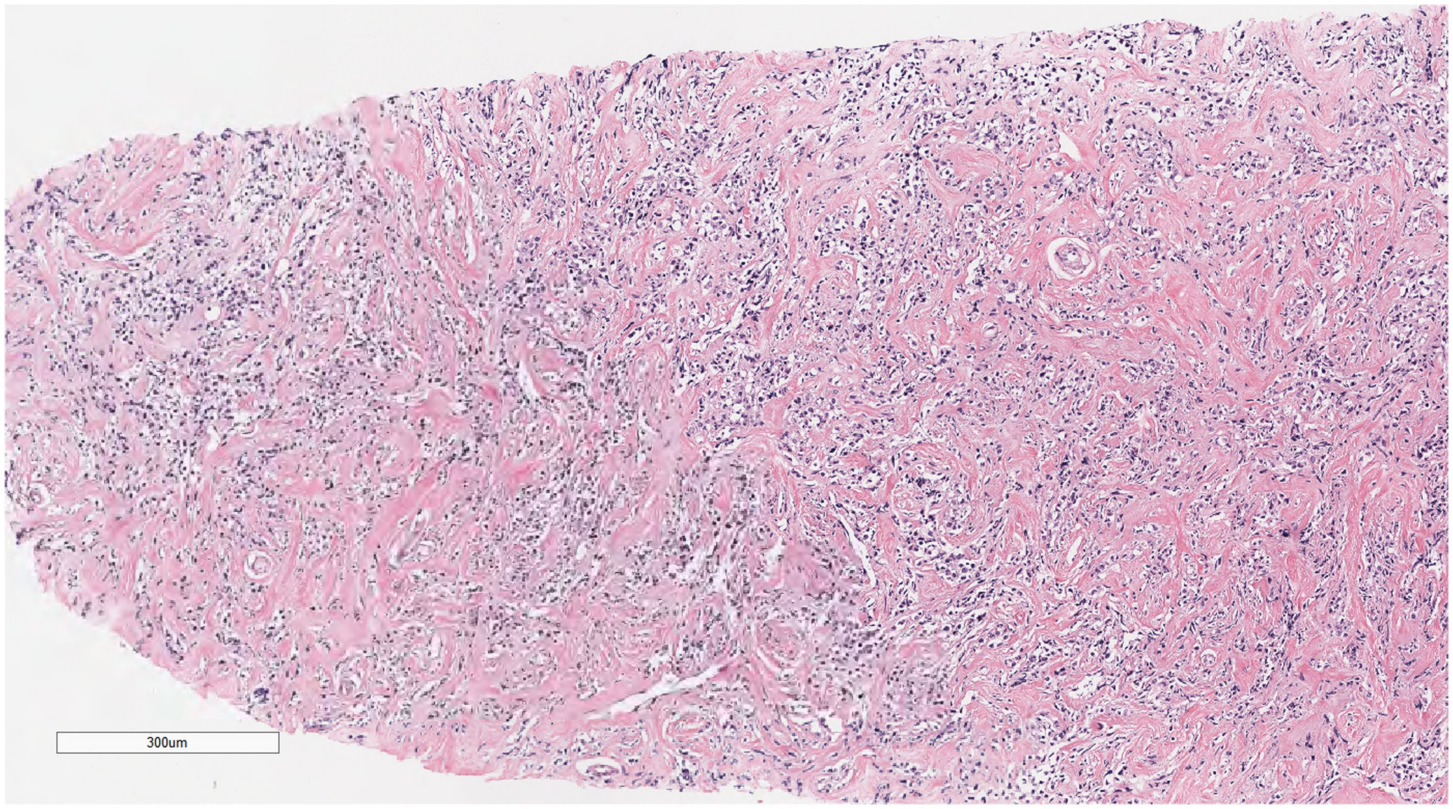
At low magnification, the tumor appears as a cord-like distribution.

**Figure 4:**
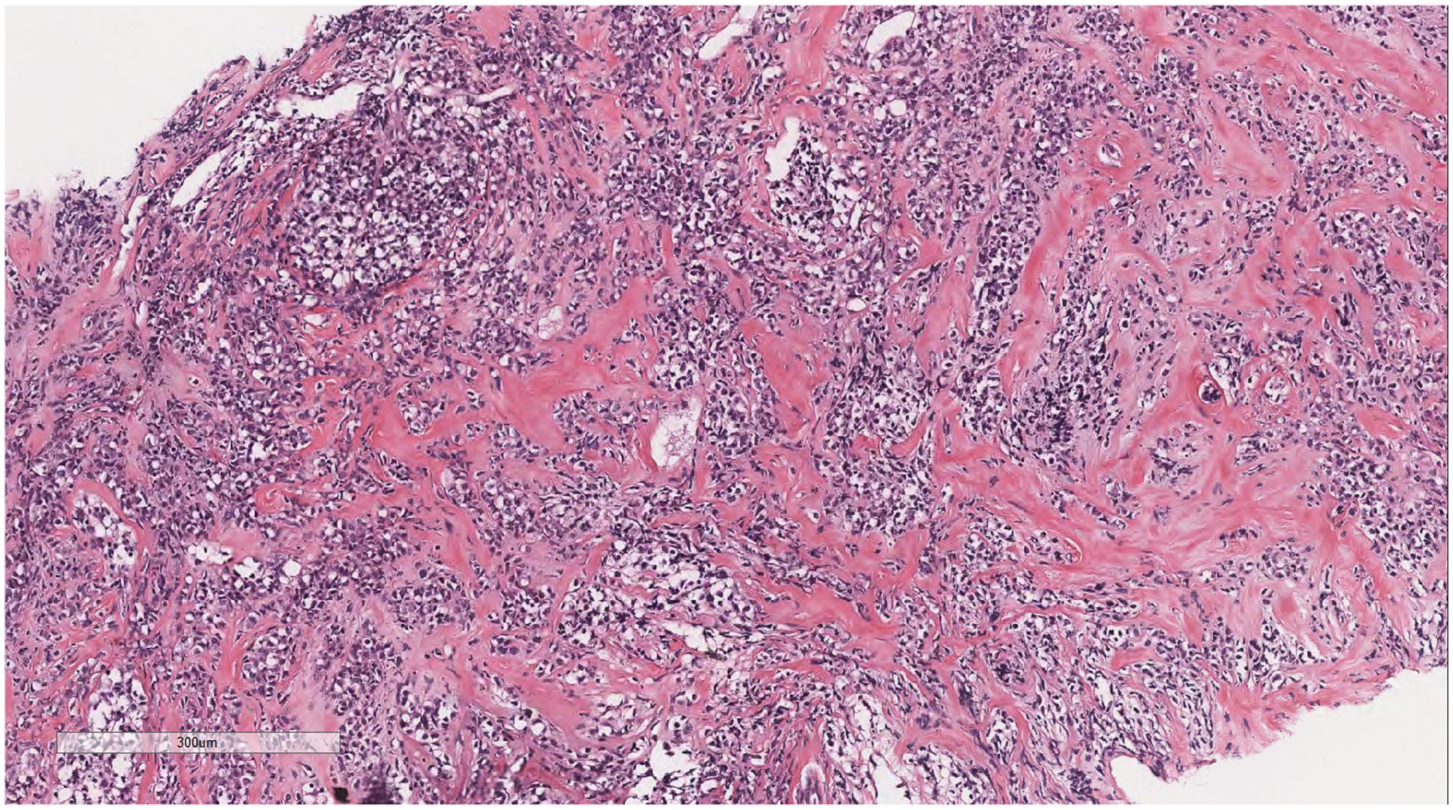
At medium magnification, the tumor cells were arranged in cords or nests and grew infiltratively into the breast stroma.

**Figure 5:**
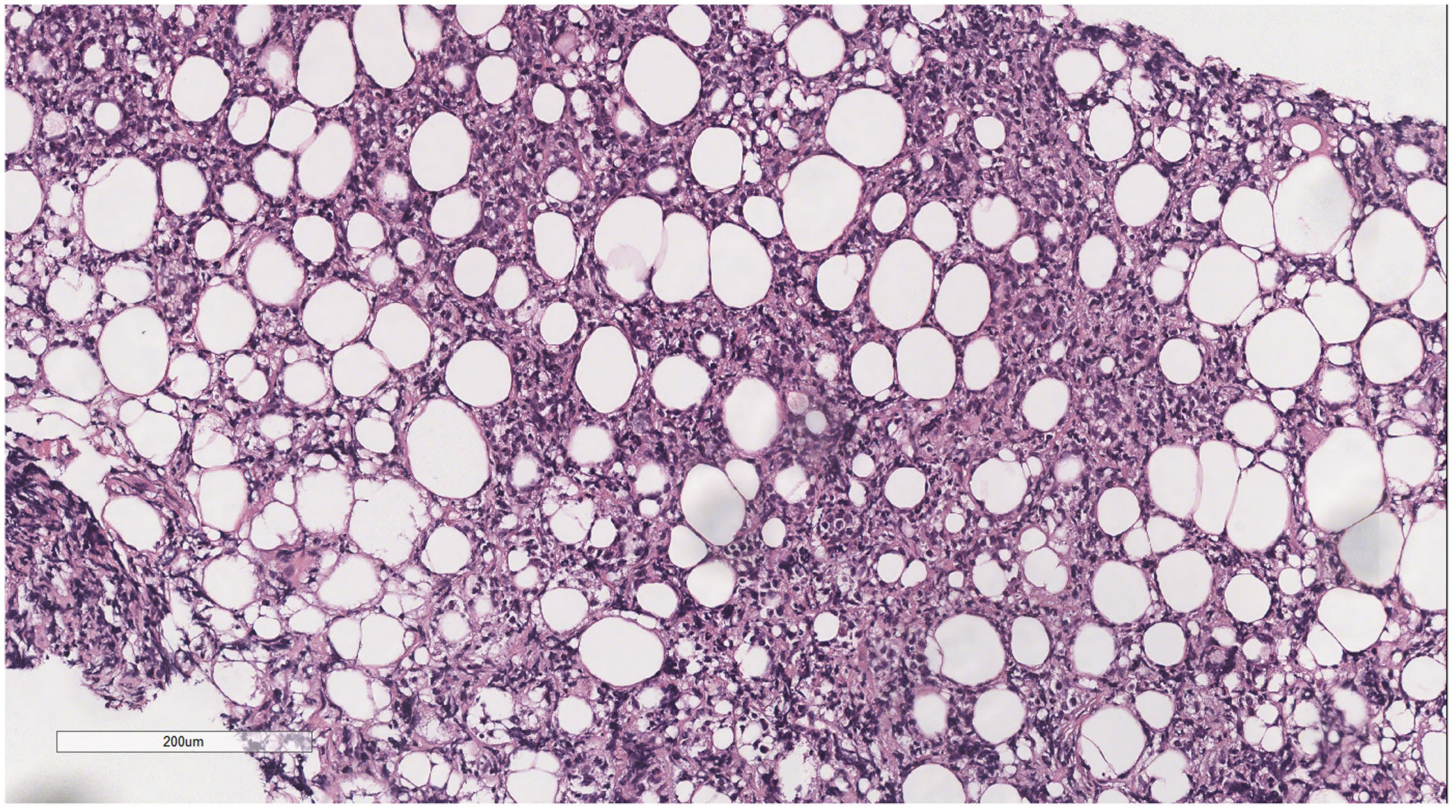
Infiltrative growth of tumor cells into fat.

**Figure 6:**
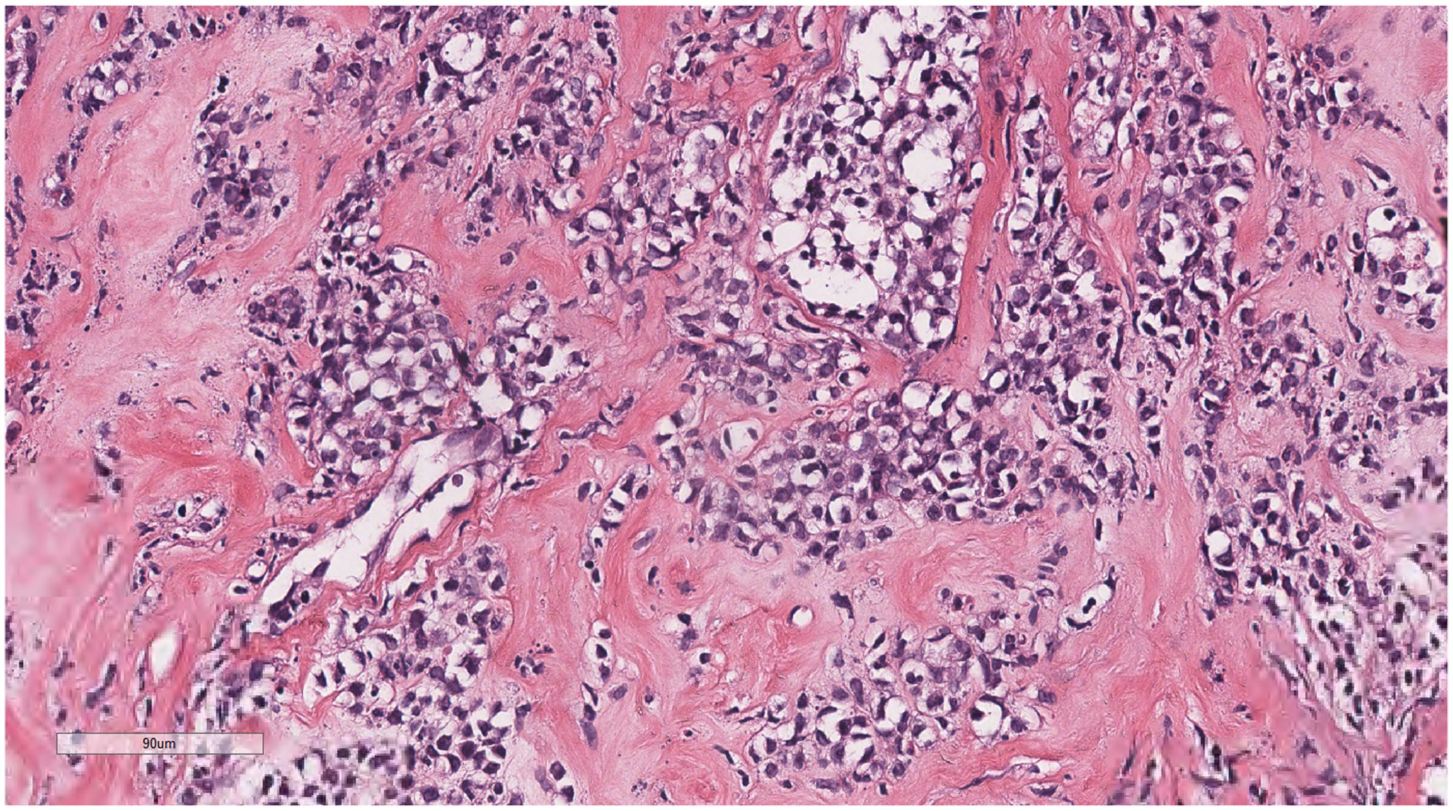
Diffusely proliferating myeloid cells, some arranged in a linear, private pattern, with slender fibrous septa.

**Figure 7:**
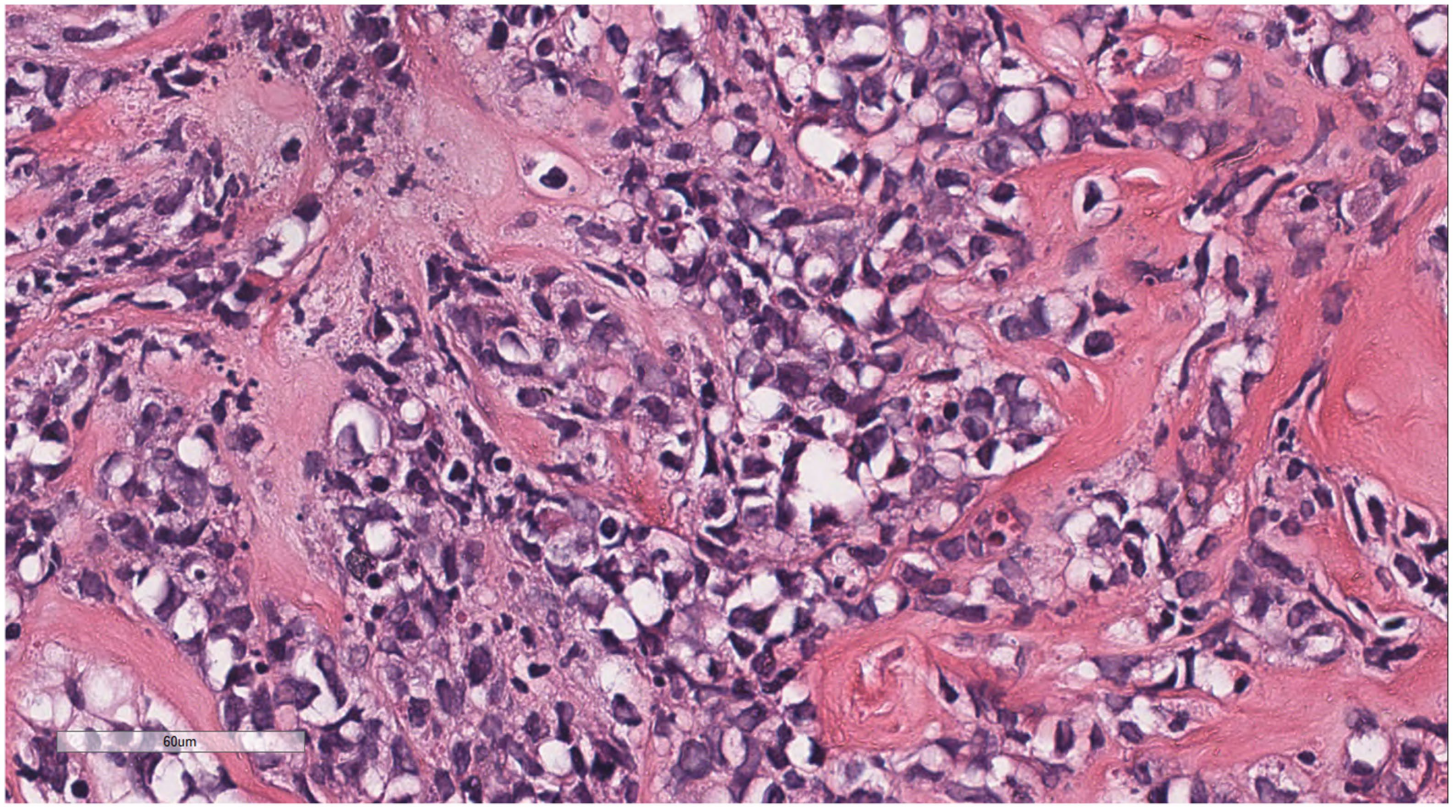
The cells were medium to large, with unclear nucleoli, the nuclei were primitive to folded, the chromatin was partially empty and bright, and there was local fibrous tissue proliferation.

**Figure 8:**
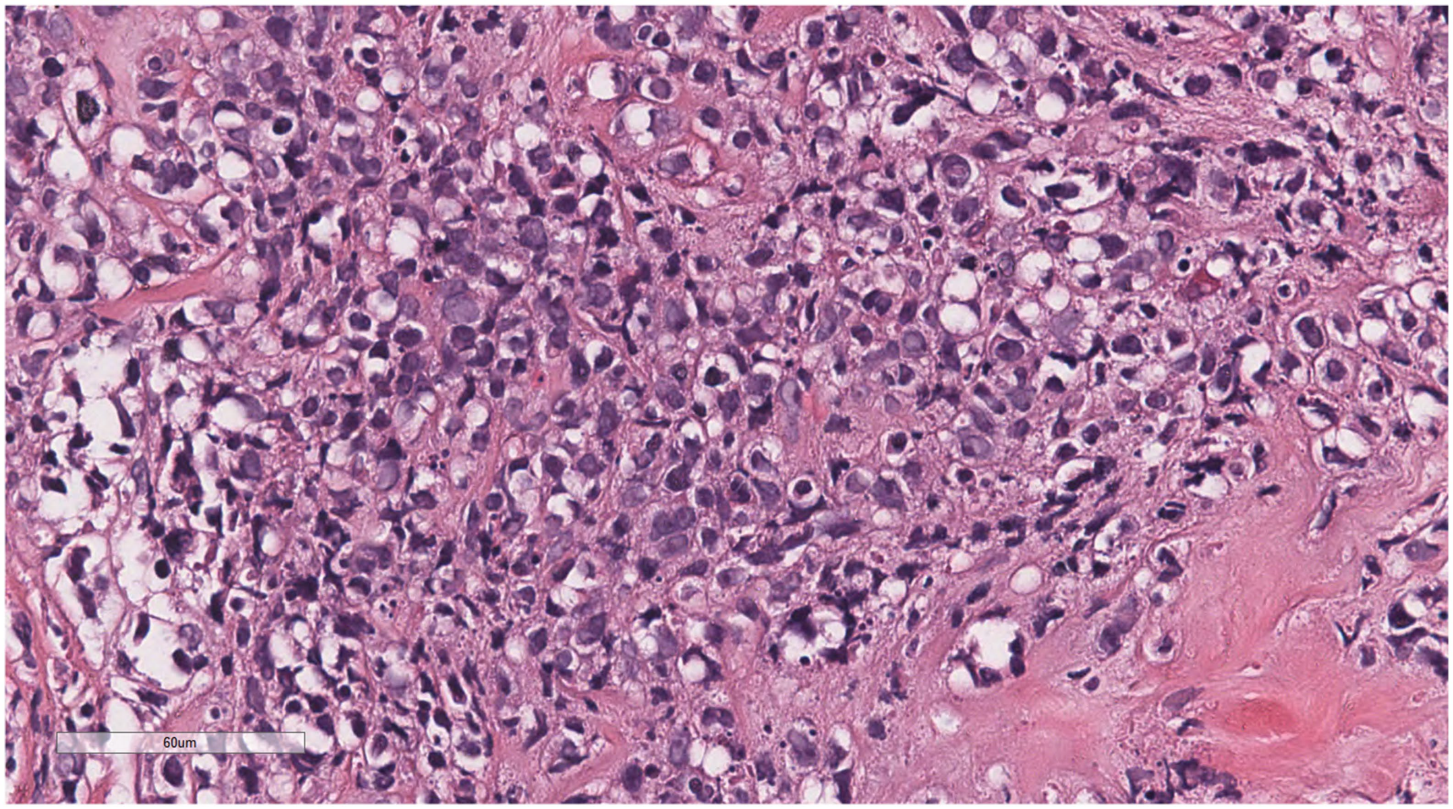
Some of the cells were medulloblastoid, with scant cytoplasm, round nuclei, and small nucleoli.

Microscopically (Figures 2–8), the breast biopsy tissue revealed diffuse proliferation of lymphocyte-like cells arranged in cords or nests, which infiltrated the breast stroma and surrounding fat. At medium magnification, patches of mononuclear cells with loose arrangements and uniform dispersion were observed. Some of these cells were arranged in a linear or nonpiled pattern, with slender fibrous septa visible. The cells were medium to large, with indistinct nucleoli, primitive to folded nuclei, fine chromatin, scattered eosinophils, and mitotic figures. Some of the cells were medulloblastoid, with scant cytoplasm, round nuclei, and small nucleoli. Some of the cells were myelomonocytic or monocytoid, with local fibrous tissue proliferation.

**Figure 9:**
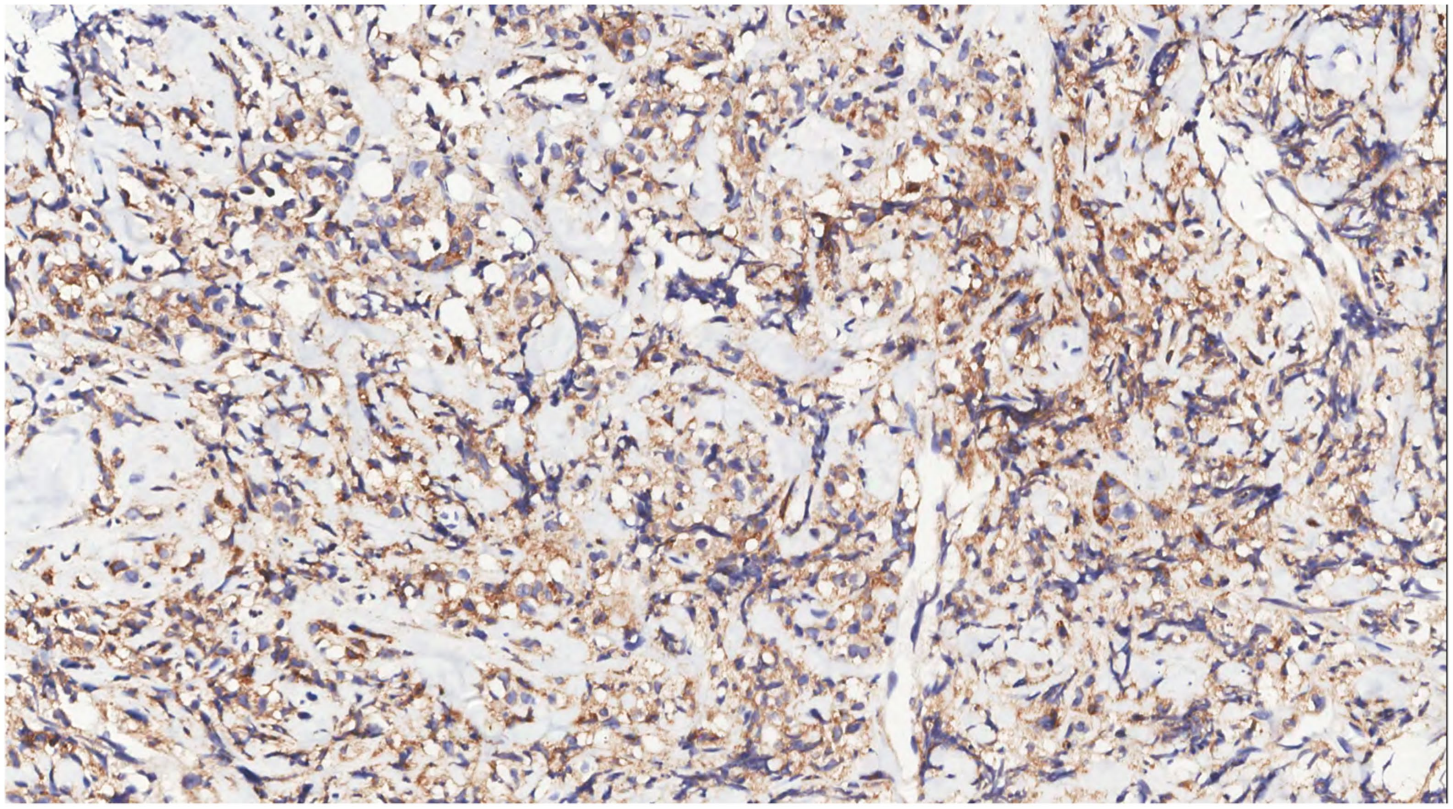
Immunohistochemistry showed LCA(+) of tumor cells.

**Figure 10:**
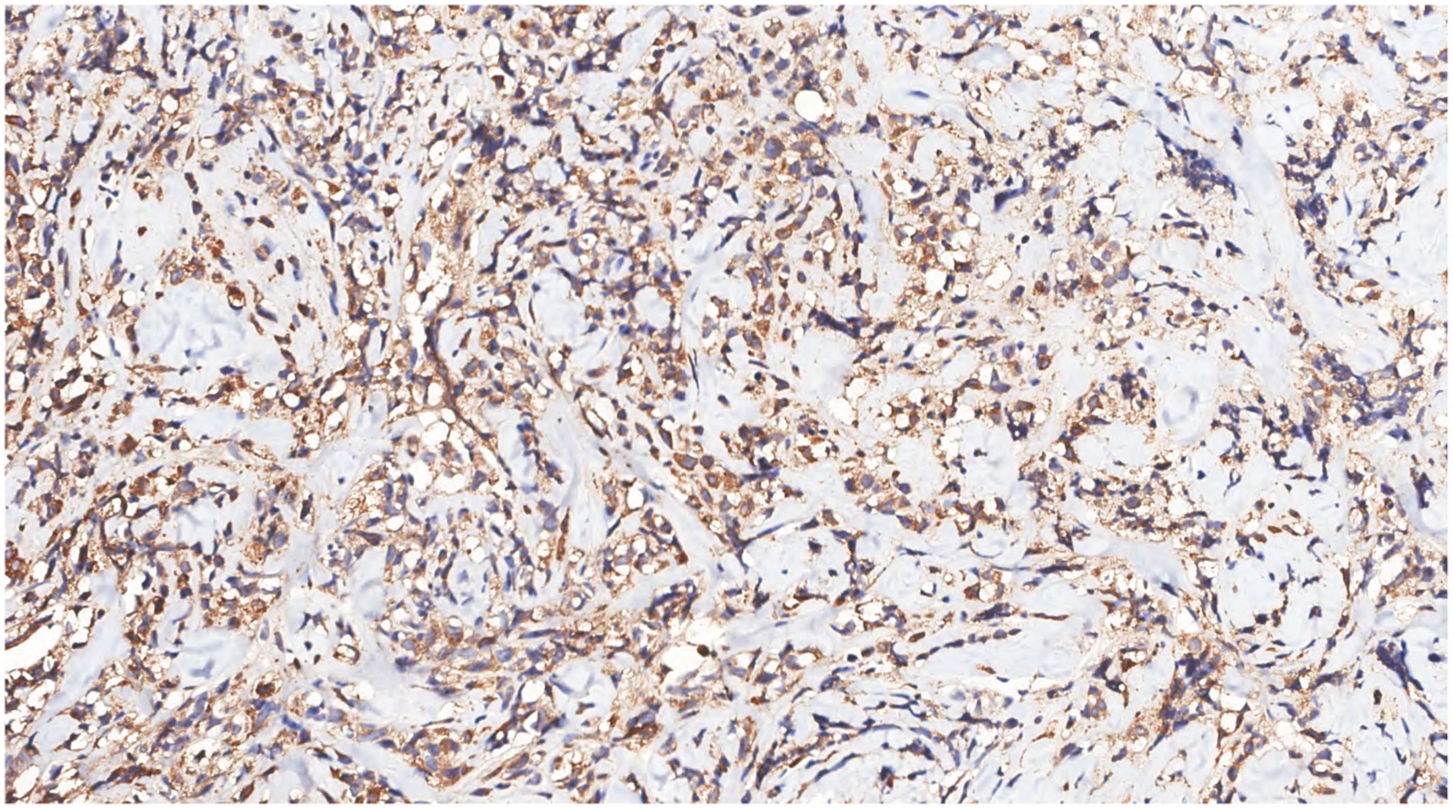
Immunohistochemistry showed MPO(+) in tumor cells.

**Figure 11:**
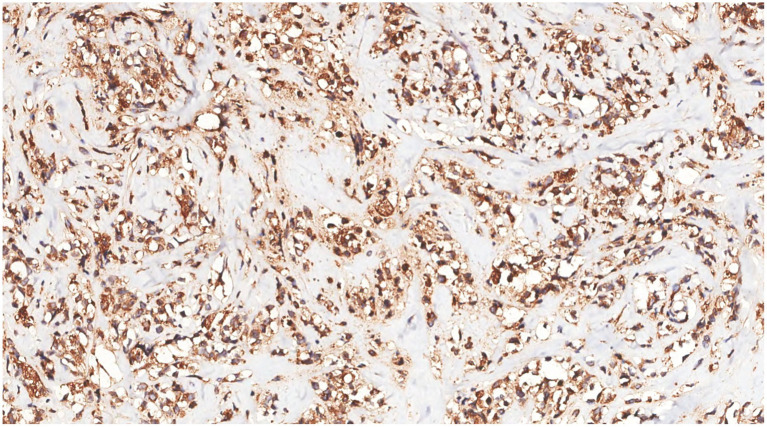
Immunohistochemistry showed that the tumor cells were CD68(+).

**Figure 12:**
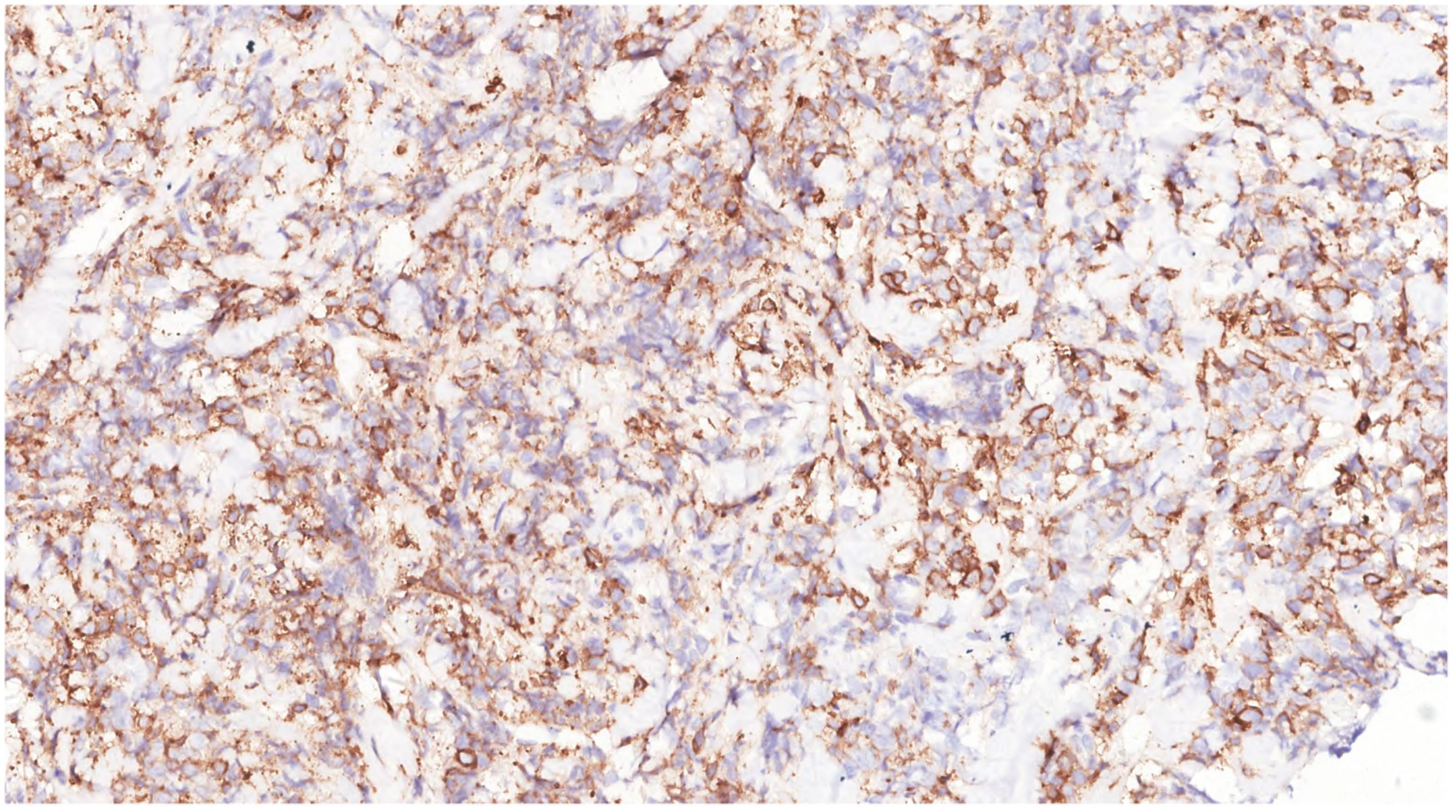
Immunohistochemistry showed that the tumor cells were CD117(+).

Immunohistochemical analysis of tumor cells revealed the following: Vimentin (+), LCA (+) (Figure 9), MPO (+) (Figure 10), CD68 (+) (Figure 11), CD117 (+) (Figure 12), CD15 (+) and CD123 (in +), CD34 (in +), CK (−), CD20 (−), CD3 (−), GATA3 (−), TRPS1 (−), and Ki-67 (70%+).

Combined with the history of AML, pathological features, and immunohistochemical results, the pathological diagnosis was MS involving the breast.

Considering the patient’s current examination results and her previous history of AML, MS of the breast due to relapse of leukemia was suspected. At the same time, bone marrow aspiration revealed blood cell morphology showing proliferation of three lineages, with myeloblasts accounting for 0.5% of the bone marrow. Minimal residual detection was performed through phenotypic analysis, with approximately 300,000 cells obtained. CD34 + cells accounted for approximately 1.65% of all nucleated cells. Primordial myeloid cells accounted for approximately 1.34% of all nucleated cells, no obvious abnormal cell population was observed, and the residual leukemic cells were less than 10–4. Based on a comprehensive consideration of the current situation, surgery was not recommended at this time. Local radiotherapy was considered the first line of treatment, with regular follow-up. The patient was followed up for 1 month, and no recurrence was found in other areas.

## Discussion

MS was first reported by Burns in 1811 and is often associated with chronic myeloproliferative neoplasms. MS of the breast is an extremely rare disease, found in only 0.12% of patients with AML (4). So far, only about 70 cases of MS of the breast have been reported in the literature. The clinical manifestations of breast MS are usually nonspecific. Although imaging plays a role, the characteristics of MS by imaging examination may resemble those of breast cancer or other malignant tumors (5). In the case of primary breast MS without a history of leukemia, the diagnosis is challenging, and the final diagnosis requires a histopathological biopsy. The clinicopathological features, differential diagnosis, treatment, and prognosis of this disease are discussed to improve the understanding of this disease.

### Clinical features

The patients’ ages ranged from 23 to 47 years (mean 33.8 years). MS mostly occurs in young people (6). Patients may have a history of AML, myelodysplastic neoplasm, or myelodysplastic syndrome, or no history of AML, presenting as isolated MS (7), and may present with elevated lactate dehydrogenase (LDH) levels. The median time from the diagnosis of AML to the development of MS of the breast was 30.6 months (range: 8 months–5.5 years). The lesion often presents as a breast mass, which may involve one or both breasts. Clinical history and elevated LDH are helpful for the diagnosis (6).

### Imaging

As one of the important auxiliary examinations for detecting MS, the manifestations of imaging examination lack high specificity, they have common characteristics, such as diffuse growth of masses, unclear boundaries, and isodensity signal (8, 9). The lesions showed significantly limited diffusion, with a marked low signal on the ADC map, and the peripheral enhancement was higher than the central (10). Most patients with MS with leukemia have abnormal bone marrow metabolism in adjacent parts or the whole body. FDG-PET scans show diffuse metabolic activity in the bone marrow cavity, while MRI may reveal abnormal bone marrow signals (11).

### Molecular genetics

Chromosome deletion, *KMT2A* rearrangement, *NPM1* mutation and *RUNX1-RUNX1T1* rearrangement can be detected in more than half of myeloid sarcoma cases (12, 13).

### Differential diagnosis

MS of the breast should be distinguished from lymphoma (14, 15), breast invasive carcinoma, lymphoblastic lymphoma/leukemia (16), poorly differentiated carcinoma and sclerosing lymphocytic lobular inflammation (17). The diagnosis can be made according to the clinical history, histomorphologic features, immunohistochemical results and molecular genetic characteristics.

### Pathological features

Microscopic examination: MS showed diffuse proliferation of medium to large myeloid cells with fine chromatin and small nucleoli (18). The immunophenotype findings were positive for LCA, MPO, CD117, CD34, CD43, CD10, lysozyme, CD68, and CD99 (6). It has been reported that MS can express B cell markers, TdT, CD123, CD4, and CD30, which may complicate the diagnosis (19). Approximately 20% of cases can express CD56; a few cases can express CD123 and CD303; and 16% of cases can express NPM1 (20).

### Treatment and prognosis

After diagnosis MS, chemotherapy was performed, or surgical resection was supplemented with chemotherapy, radiotherapy, and donor lymphocyte infusion (DLI) (21). Some researchers have suggested that, in addition to the abovementioned treatment options for MS relapsed after allogeneic hematopoietic stem cell transplant (allo-HSCT), donor lymphocyte infusion (DLI) and a second bone marrow transplantation should also be considered (22, 23).

## Conclusion

In conclusion, the clinical manifestations of breast MS are typically nonspecific. Although imaging plays a role in diagnosis, the imaging characteristics of MS can resemble those of breast cancer or other malignant tumors, making the diagnosis of primary breast MS without a history of leukemia challenging. For patients with a history of AML, regular review of imaging indicators is essential for early detection, diagnosis, and treatment, which can help improve the prognosis.

## Data Availability

The original contributions presented in the study are included in the article/supplementary material, further inquiries can be directed to the corresponding author/s.
